# Circadian regulation of the *Drosophila* astrocyte transcriptome

**DOI:** 10.1371/journal.pgen.1009790

**Published:** 2021-09-20

**Authors:** Samantha You, Alder M. Yu, Mary A. Roberts, Ivanna J. Joseph, F. Rob Jackson

**Affiliations:** 1 Department of Neuroscience, Tufts Graduate School of Biomedical Sciences, Tufts University School of Medicine, Boston, Massachusetts, United States of America; 2 Department of Biology, University of Wisconsin–La Crosse, La Crosse, Wisconsin, United States of America; Universidad de Valparaiso, CHILE

## Abstract

Recent studies have demonstrated that astrocytes cooperate with neurons of the brain to mediate circadian control of many rhythmic processes including locomotor activity and sleep. Transcriptional profiling studies have described the overall rhythmic landscape of the brain, but few have employed approaches that reveal heterogeneous, cell-type specific rhythms of the brain. Using cell-specific isolation of ribosome-bound RNAs in *Drosophila*, we constructed the first circadian “translatome” for astrocytes. This analysis identified 293 “cycling genes” in astrocytes, most with mammalian orthologs. A subsequent behavioral genetic screen identified a number of genes whose expression is required in astrocytes for normal sleep behavior. In particular, we show that certain genes known to regulate fly innate immune responses are also required for normal sleep patterns.

## Introduction

Circadian clocks regulate physiology, cognition, behavior, and metabolism. In animals with a centralized nervous system, circadian clocks are localized in both neurons and glia of the brain [1–9] These clocks utilize transcriptional/translational feedback loops (TTFLs) in which clock genes promote their own oscillations by activating or inhibiting transcription/translation of other members of the loops [10]. Importantly, there are downstream clock-controlled genes (CCGs) in all cells examined, many of which regulate behavior. CCGs relevant for behavior have been identified in circadian profiling studies, but most such studies have not differentiated between cell types and thus might have missed “cycling genes” with a limited tissue or cell-specific (e.g., astrocyte) expression pattern [However, see [11–14]].

Translating ribosome affinity purification (TRAP) was developed as a method of profiling ribosome-associated RNAs in a cell-type-specific manner [15,16]. Using this method, a ribosomal subunit (L10a) tagged with enhanced green fluorescent protein (EGFP) is expressed in a cell type of interest. Ribosome-bound RNAs are then immunoprecipitated using a high-affinity EGFP antibody and identified using RNA-seq methods. Although not all ribosome-bound transcripts are actively translated, for the majority TRAP methods provide a snapshot of RNA transl**a**tion. We previously adapted this technology for use in *Drosophila* given the existence of the Gal4/UAS binary expression system and the ease with which cell-specific expression studies can be carried out. TRAP permits simple isolation of ribosome-bound RNAs without the need for tissue dissection or cell sorting, thereby providing a more accurate representation of *in vivo* gene expression. Our previous studies, for example, have documented circadian translational profiles of *Drosophila* clock cells [17].

In the present study, we carried out TRAP-seq analyses to identify *Drosophila* CCGs within astrocytes of the adult brain. Such an analysis has not been carried out in any biological model. Mammalian and *Drosophila a*strocytes are morphologically and functionally similar [18–20] and have conserved genome-wide expression profiles [21]. This cell type has important functions in circadian behavior and sleep [3,19,21–27]. Although it is known that astrocytes and other glial cell types possess circadian clocks, little is known about the complement of astrocyte genes showing circadian changes in expression, nor have their behavioral functions been studied. Thus, we employed TRAP by using EGFP-L10a expression in astrocytes to identify ribosome-bound RNAs showing circadian changes in abundance. This analysis revealed RNAs representing 293 genes–many of them previously unidentified–that cycle in abundance in fly astrocytes. Using this list as a starting point, we carried out an RNA-based genetic screen to detect genes required in glial cells for normal patterns of fly sleep. Of interest, we discovered that certain innate immune signaling components are required for normal sleep.

## Results

### TRAP analysis to identify circadianly regulated astrocyte mRNAs

**Fig 1:**
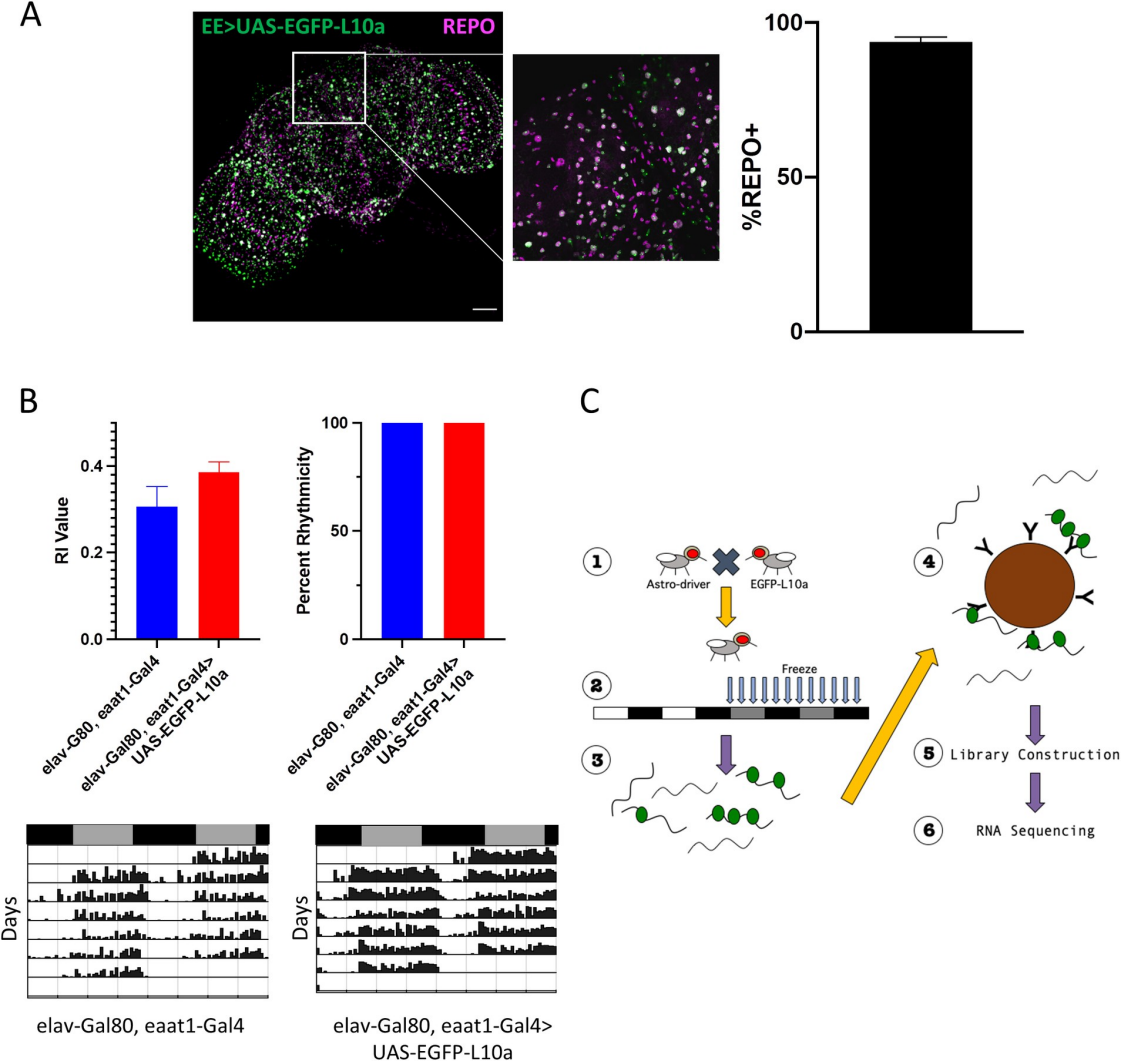
Using TRAP for circadian profiling of fly astrocytes. A) Eaat1-Gal4 with elav-Gal80 (EE) was used to express EGFP-L10a in astrocytes. Brains were also stained with REPO (magenta) to visualize glial cells. Maximum-projection image (left) shows overall distribution of both EGFP-L10a and REPO throughout the brain at 20X magnification. The offset image shows colocalization of EGFP-L10a with REPO at high-magnification (60X). Scale bar = 50 μm. EGFP-L10a cells were counted for REPO positivity with 93.7% ± 1.7% EGFP-L10a cells positive for REPO (n = 4 brains). B) Representative actograms of DD behavior for data represented in C. Black bars represent night (CT0-11) while gray bars represent day (CT12-23). C) Free-running (DD) behavior data for elav-G80, UAS-EGFP-L10a (blue) and elav-Gal80, eaat1-Gal4>E2 (red). Expression of EGFP-L10a does not result in decreased circadian rhythmicity. n = 10 flies for each group. D) Flies were crossed to make progeny that expressed EGFP-L10a in astrocytes (1) and then entrained to a 12:12 LD schedule. Flies were flash frozen every four hours across 2 days in DD (2). RNA was extracted (3) and immunoprecipitated (4) using a high-affinity EGFP antibody. RNA was then converted into cDNA libraries (5) and processed for sequencing (6).

We employed Translating Ribosome Affinity Purification (TRAP) to identify ribosome-bound mRNAs that exhibit circadian rhythms in abundance in fly astrocytes. For this analysis, we generated flies that expressed a tagged large ribosomal subunit (L10a) [17] in astrocytes using the Gal4/UAS binary system and the eaat1-Gal4 driver. As this driver also expresses in a few T1 lamina neurons of the visual system [28,29], we included elav-Gal80 in the genetic background to inhibit Gal4 activity in neurons. Crosses were performed as described in the methods to generate adults that express EGFP-L10a predominantly in astrocytes. As expected, the EGFP-L10a signal was observed in some but not all glial cells of the adult brain, as assessed by antibody staining for EGFP and REPO, a pan-glial protein (Fig 1A). Of cells marked by EGFP-L10a, the great majority were REPO-positive. As previously reported, we see expression of the tagged subunit in the cytoplasm along with the nucleoli, as expected if the subunit incorporated into ribosomes [17]. To ensure that the EGFP-L10a incorporation in astrocytes did not adversely affect circadian behavior, we examined free-running rhythms via locomotor activity and found no effect compared to the control (Fig 1B and 1C).

To generate a circadian translatome for astrocytes, flies expressing EGFP-L10a in astrocytes were collected in four-hour intervals during the first 48 hours of constant darkness (DD) after entrainment to a regular 12 hours lights on, 12 hours lights off (LD 12:12) schedule (Fig 1D). Ribosome-bound RNA was isolated from head tissues by immunoprecipitation using a high-affinity EGFP antibody. This procedure was repeated with flies emerging from independent crosses in order to generate two biological replicates. cDNA libraries were generated from the duplicate samples for each time point for RNA-seq analysis.

Single-end, 100-base pair reads were obtained for cDNA libraries using an Illumina HiSeq2000 instrument, with an average of >20 million reads per sample. Read quality was assessed and reads were aligned to the Berkeley Drosophila Genome Project Release 6 genome assembly as described in Materials and Methods (mapping statistics shown in S1 Table). Importantly, the reads representing biological replicates for each time point were similar to one another with the correlation coefficient (r) **≥** 0.86 and averaging 0.90 across the first day of DD. Overall across both days, the (r) **≥** 0.74 and averaged 0.85 (S1 Fig). Collectively across all time points, we detected translating mRNAs representing 7802 genes (S2 Table). As expected for astrocytes, the list of genes includes *gabat*, *Gs2*, *eaat1*, *ebony*, *Nkt*, and *alrm* among others known to be expressed in this cell type.

### Expression profiles derived using eaat1-Gal4 and alrm-Gal4 are largely overlapping

**Fig 2:**
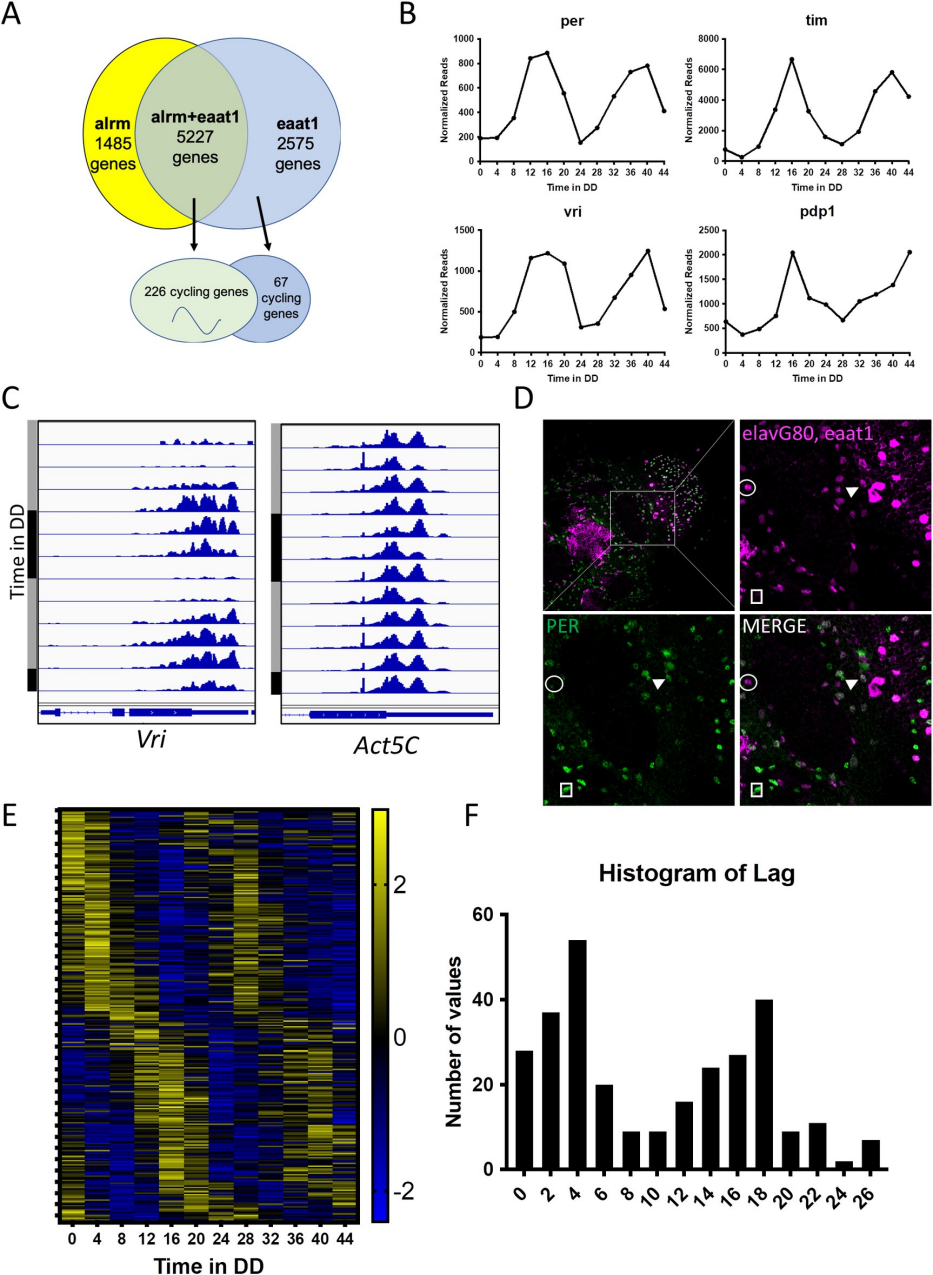
TRAP identifies rhythmic ribosome binding (translation) in astrocytes. A) Comparison of profiled genes in the current eaat1-Gal4 generated study versus a previous alrm-Gal4 generated study [30]. Yellow represents alrm-Gal4 study, blue represents the current eaat1-Gal4 study and green represents the overlap genes. B) Translational profile of select clock genes with the y-axis representing average normalized read counts and the x-axis representing hours in DD. C) Visualization of circadian cycling in sequence reads for the clock gene *vri* and the housekeeping gene *Act5C*. Black bars represent night (CT0-11) whereas gray bars represent day (CT12-23). The relevant intron-exon organization for each gene is shown at the bottom of the panels. D) Confirmation of astrocytic expression of PER by using elav-Gal80, eaat1-Gal4 to drive the nuclear marker DsRed in astrocytes (magenta) and staining with PER (green). Dissections were performed at CT0. Circle represents non-PER+ astrocyte. Square represents PER+ non-astrocyte. Arrows represent PER+ astrocytes. Scale bar = 50 μm. E) Heat map showing normalized levels (z-score) of translation across two days in DD for 293 cycling genes. Yellow represents highest translation whereas blue represents lowest translation. F) Histogram of all 293 cycling genes shows two peaks for translation at CT4 and CT18.

Our previous astrocyte-profiling experiments were performed using the alrm-Gal4 driver [21,30] which is fairly specific for astrocytes [31] though also weakly expressed in ensheathing glia [32]. We chose eaat1-Gal4 for the present studies because it is expressed at higher levels than alrm-Gal4, facilitating the TRAP immunoprecipitation procedure. The eaat1-Gal4 expression pattern is predominantly astrocytic but also includes some cortex glia [29]. Given these reported differences in expression patterns, we were curious about overlap between the two astrocyte predominant expression profiles. As shown in Fig 2A, the overlap of the two profiles includes 5227 of the 7802 genes detected in our circadian TRAP analysis, suggesting the majority of genes should be considered high confidence astrocyte genes. This represents 78% of genes in the alrm-Gal4 experiments and 67% of the genes in the eaat1-Gal4 experiments. In addition, we found that 226 of 293 identified cycling RNAs (see below) are co-expressed with alrm and eaat1 (Fig 2A). We note that genes differentially expressed in alrm- or eaat1-containing cells may also reflect expression drivers in heterogeneous astrocyte populations rather than another type of glia; alrm-Gal4, for example, apparently does not express in all astrocytes [33]. An important caveat of this comparison is that we only examined gene expression using alrm-Gal4 at a single time point (CT1) and certain genes may be expressed at low levels at this time of day.

### Astrocyte-specific TRAP profiling detects circadian rhythms in ribosome binding

The algorithm JTK-CYCLE was used to detect circadian rhythms in ribosome associated RNAs [34]. This algorithm calculates period, phase and amplitude of cycling transcripts, and it detected 293 mRNAs with significant (p<0.05) circadian changes in ribosome association (S3 Table). Of the 293 cycling mRNAs detected using eaat1-Gal4, 226 of them were also detected using alrm-Gal4, including all the aforementioned rhythmic clock genes. This set can be considered “high-confidence” astrocyte cycling genes. Finally, 50 of the 293 transcripts were previously identified as showing “enriched” expression in fly astrocytes compared to total head RNA [21,30]. Glia are known to contain PER-based oscillators [8], and importantly, known rhythmic clock genes *per*, *tim*, *vri*, *and pdp1* were among the cycling collection (Fig 2B and 2C). In contrast, *Clk* did not exhibit robust and statistically significant circadian changes in these experiments, although there was obvious low-amplitude cycling with examination of the sequence reads for this gene (not shown). This may reflect failure of JTK.CYCLE to detect cycling for mRNAs with a low expression level, heterogeneity for *Clk* expression in the astrocyte population or (perhaps less likely) a difference between neuronal and glial oscillators.

A previous report indicated that that fly astrocytes do not express PER [35], but we have identified astrocytic PER in this study and in previous studies (Fig 2B and 2D) [21]. In agreement, another recent study examined PER localization specifically in the distal medulla of the optic lobe and found that astrocytes in that area contain either low or undetectable amounts PER [36]. Using the same astrocyte driver as our TRAP experiments (eaat1-Gal4 with elav-Gal80), we marked astrocytes with nuclear DsRed (magenta) and stained the brains with PER (green) (Fig 2D). We found DsRed labeled astrocytes both with and without PER (30.3% ± 3.7% contained the clock protein; n = 5 brains), indicative of heterogeneity in fly astrocytes.

### Daily peaks of ribosome-binding indicate two dominant phases of translation

A previous study from our lab identified genes showing rhythms of ribosome-binding in circadian clock cells [17]. That study indicated there were two major phases of rhythmic ribosome binding (translation), one at mid-day and one during the night. These time periods are predicted to correspond to periods of lower metabolic expenditure due to low locomotor activity. Similarly, we observed two major peaks of ribosome binding for astrocytes (Fig 2E and 2F). The previous circadian TRAP study utilized the tim-Gal4 driver, which is expressed in both neurons and glial cells [17], but there was surprisingly little overlap between the lists derived in Huang et al. and the current study. Out of 293 genes identified in the current study, only 34 were previously determined to be cycling in tim-containing clock cells. The lack of overlap is likely due to the cell-type specificity of the two profiling studies; i.e, certain genes that cycle in astrocytes may not do the same in *tim*-positive cells. It is of interest, nonetheless, that daily profiles of ribosome binding are similar in clock cells and astrocytes.

### Categories of cycling genes

We performed gene ontology (GO) analyses using Database for Annotation, Visualization and Integrated Discover (DAVID) to define overrepresented categories of proteins encoded by cycling genes [37]. Twenty-nine biological processes were determined to be enriched, the majority of which are contained within two more general categories: “Stress and Immunity” and “Circadian and Sleep” (Table 1). Thirteen members of the cytochrome P450 family were present in our “Stress and Immunity” list. These could be separated into two groups for which peak translation was either at the very beginning of the day (ZT0) or in the middle of the night (ZT16) (S2A Fig).

**Table 1:** Gene Ontology for Enriched Biological Processes.

Term	Count	%	P-Value	Fold Enrichment
oxidation-reduction process	24	8.2	1.50E-05	2.8
circadian rhythm	9	3.1	3.90E-05	7
rhythmic behavior	4	1.4	2.20E-03	14.8
response to DDT	5	1.7	2.50E-03	8.6
response to oxidative stress	8	2.7	3.00E-03	4.2
modulation of synaptic transmission	5	1.7	8.40E-03	6.2
response to fungus	4	1.4	8.90E-03	9.1
proteolysis	19	6.5	1.00E-02	1.9
aromatic amino acid family metabolic process	3	1	1.10E-02	18
insecticide catabolic process	4	1.4	1.10E-02	8.4
response to bacterium	5	1.7	1.50E-02	5.2
innate immune response	7	2.4	2.10E-02	3.2
entrainment of circadian clock	3	1	2.50E-02	12
mating behavior	3	1	2.90E-02	11.1
carbohydrate phosphorylation	3	1	2.90E-02	11.1
developmental pigmentation	3	1	3.30E-02	10.3
response to endoplasmic reticulum stress	4	1.4	3.50E-02	5.5
cellular response to heat	3	1	3.80E-02	9.6
negative regulation of transcription regulatory region DNA binding	2	0.7	4.10E-02	48
sleep	7	2.4	4.10E-02	2.8
visual behavior	3	1	4.20E-02	9
locomotor rhythm	5	1.7	4.40E-02	3.8
regulation of circadian sleep/wake cycle, sleep	3	1	4.70E-02	8.5
UDP-N-acetylglucosamine metabolic process	2	0.7	6.10E-02	32
cuticle pigmentation	3	1	6.40E-02	7.2
carbohydrate metabolic process	6	2.1	6.90E-02	2.7
circadian temperature homeostasis	2	0.7	8.00E-02	24
neuron cellular homeostasis	3	1	9.40E-02	5.8
chitin catabolic process	3	1	9.40E-02	5.8
**Stress and Immunity**	**Circadian and Sleep**			
Sardh, Per, bgm, Cyp6a17, Ple, nec, TotA, CG4842, Drat, Cyp6a23, SPE, Hf, Cyp18a1, sud1, Cyp6a21, Thor, Cyp6a8, Cyp305a1, Hpd, Cbs, Shop, Naprt, Ire1, Cyp4p1, CG1434, Cyp28d1, Meigo, CG10211, PGRP-LB, TotX, Sid, TotC, CG13077, CG9372, Cyp12d1-p, Cyp4d21, Cyp309a1, Cyp6t1, CG9747, Tg, Tsf1, Cyp4aa1	qsm, per, bgm, Lttn1, CG11407, CG7079, to, vri, Drat, CG5945, cry, Acer, Spn27A, Pdp1, CG2016, tim			

We also performed a literature search to assess what was known about each cycling astrocyte gene. This indicated that only 52 were previously identified as having circadian expression profiles (S4 Table). Thus, the majority identified in the present study are newly described cycling genes. Among this collection are genes of the Turandot family (S2B Fig), known to be induced by stress and innate immune responses via the JAK-STAT pathway [38,39]. Of related interest, mRNAs encoding proteins of the Bomanin and Growth Blocking Peptide families–also induced by stress and immune responses–show circadian rhythmicity (Figs 4 and S2B). Similarly, multiple components of Toll signaling, *necrotic* (*nec*), *Serpin 27A* (*Spn27A*), and *Spätzle-processing enzyme* (*spe*), also show rhythmicity in fly astrocytes (S2D Fig). *Spe* cleaves Spätzle (SPZ) protein to generate a Toll receptor ligand. A recent study identified astrocytic *spz* as essential for communicating sleep need via neuronal Toll signaling [40].

### Identification of genes required for normal sleep

A number of the observed cycling astrocyte genes are regulated by sleep deprivation or associated with sleep defects (e.g., *Bgm* and *Acer*). As proof of principle that other cycling mRNAs might be important for this rhythmic process, we performed RNAi-based screens to identify additional genes important for sleep behaviors. Certain genes were screened using the repo-Gal4 driver to produce knockdowns but most were screened using eaat1-Gal4, a driver expressed predominantly in astrocytes (S5 Table).

**Fig 3:**
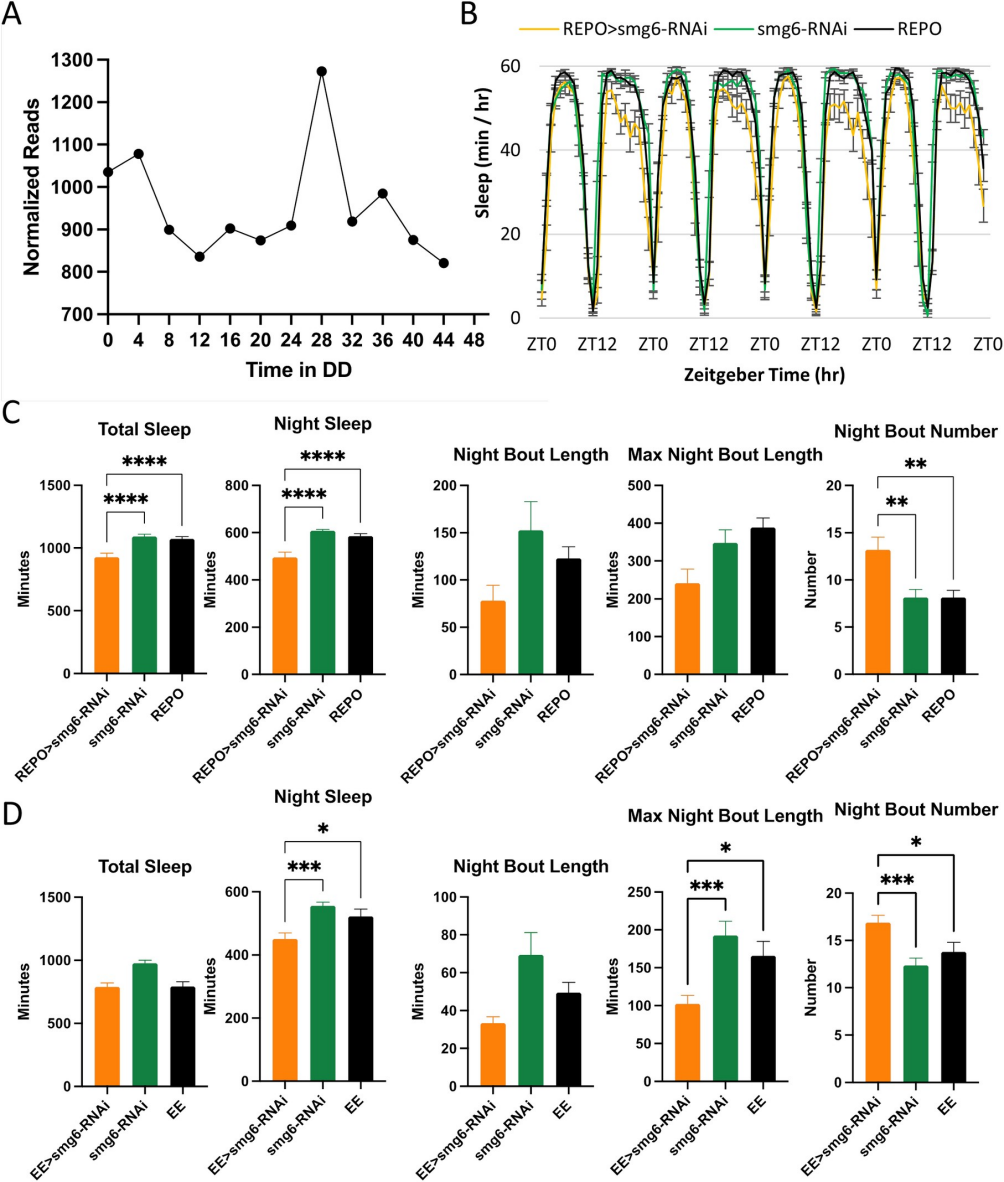
Smg6 is required for normal night sleep. A) Normalized read count across two days in DD derived from the astrocyte TRAP. Results are averages of two data sets per time point. B) Average sleep behavior across four days for REPO>SMG6-RNAi (orange, n = 23), SMG6-RNAi (green, n = 25), and REPO (black, n = 32). C) Average total sleep, night sleep, night bout length and night bout number for REPO (pan-glial) experiments. Results are mean ± SEM, One-way ANOVA, **p < 0.01, ****p < 0.0001. D) Average total sleep, night sleep, night bout length, and night bout number for EE (astrocyte) experiments. Results are mean ± SEM, One-way ANOVA, *p < 0.05, ***p < 0.001.

As post-transcriptional mechanisms are known to regulate rhythmicity, we examined behavior in flies with a knockdown of *smg6*, which encodes a nuclease required for nonsense-mediated decay (NMD). *Smg6* mRNA exhibited cycling across 48 hours of DD with peak abundance during the morning (Fig 3A). Knockdown of *Smg6* in all glial cells significantly decreased night sleep and increased the number of night sleep bouts (Fig 3B and 3C), indicating sleep fragmentation. As expected for those results, the length of night sleep bouts was decreased but not to statistical significance. There was no effect on day sleep (S3A Fig). We tested a second smg6 RNAi transgene but did not observe any significant effects on night sleep, an uninterpretable negative result. As our profiling experiment focused specifically on astrocytes, we repeated these experiments with the eaat-Gal4 driver and found similar results (including a significant decrease in the average maximum night bout length), suggesting an astrocytic specific role for SMG6 in night sleep (Figs 3D and S3B).

Given the presence of many cycling immune- and stress-related mRNAs in our dataset, we screened a number of the relevant genes (S5 Table) for sleep phenotypes. For example, we examined the function of the Turandot (Tot) family of genes, which are induced with stress. Knockdown of *TotA* decreased day sleep amount with a concomitant decrease in day sleep bout length and increased day bout number (S4A and S4B Fig). Similarly, knockdown of *TotC* in astrocytes caused day and night sleep fragmentation (S4C and S4D Fig).

**Fig 4:**
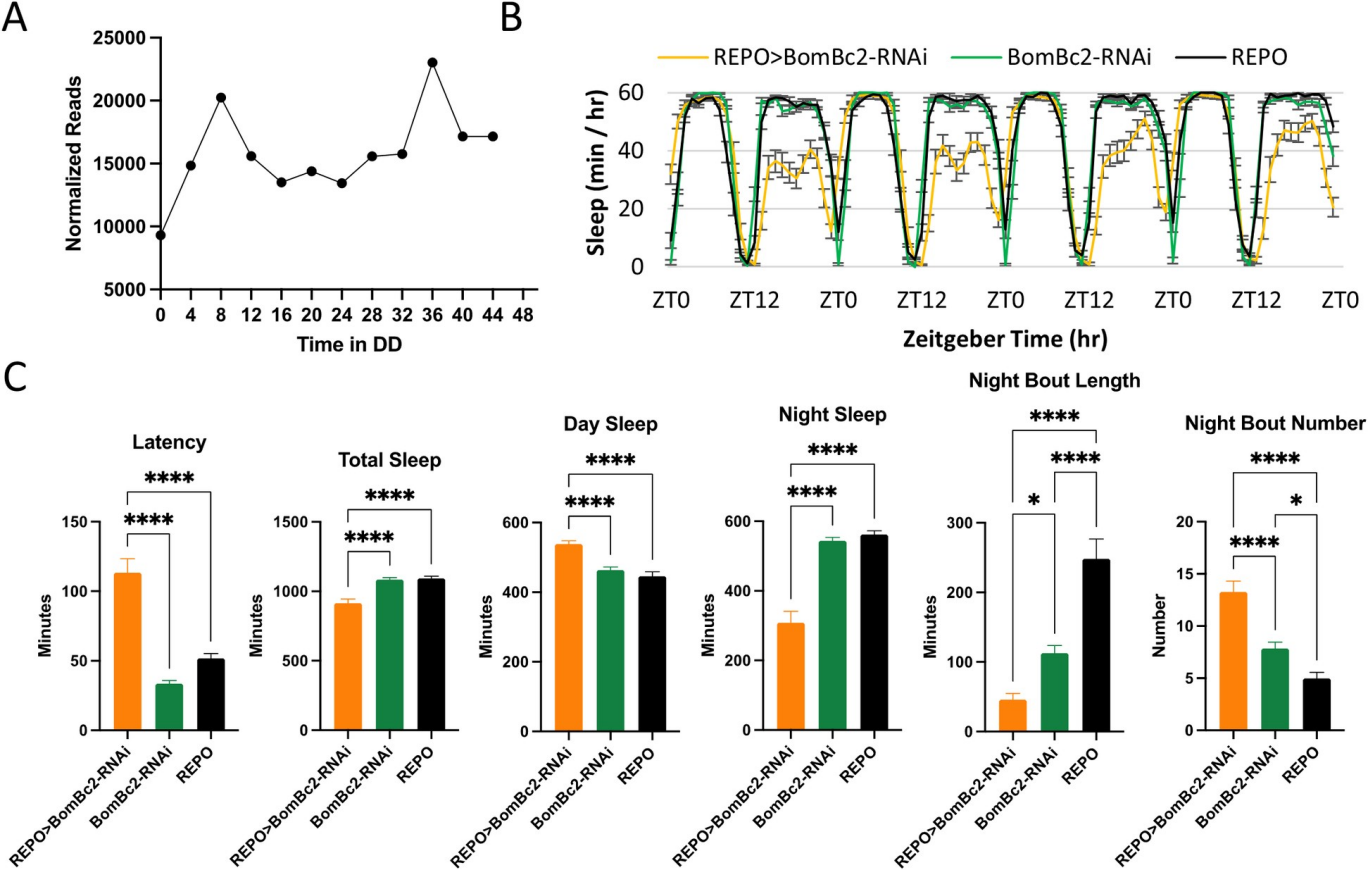
BomBc2 is required in glia for normal sleep behavior. A) Normalized read count across two days in DD derived from the astrocyte TRAP. Results are averages of two data sets per time point. B) Average sleep behavior across three days for REPO>BomBc2-RNAi (orange, n = 30), BomBc2-RNAi (green, n = 30), and REPO (black, n = 30). C) Average latency, total sleep, day sleep, night sleep, night bout length, and night bout number. Results are mean ± SEM, One-way ANOVA, *p < 0.05, ****p < 0.0001.

BomBc2, also identified as a cycling astrocyte gene (Fig 4A), is a member of a family of small, secreted immune induced peptides called Bomanins (Boms). Knockdown of BomBc2 in glia resulted in significantly decreased night sleep and increased day sleep, particularly in the first half of the day (Fig 4B and 4C), with an expected increase in sleep latency (Fig 4C). Characteristic of many sleep mutants, sleep was significantly fragmented with shorter and more frequent night sleep bouts (Fig 4C). The knockdown flies also exhibited increased day sleep, although this did not fully compensate for the decrease observed during the night (Fig 4B). Expression of a second *BomBc2* RNAi resulted in similar phenotypes although the sequence of that RNAi partially overlaps the one employed in Fig 4. Surprisingly, knockdown of *BomBc2* using the eaat1-Gal4 driver did not result in sleep alterations, suggesting that eaat1-Gal4 does not produce a sufficient knockdown of BomBc2 or that the gene functions in a non-astrocyte class of fly glial cells.

**Fig 5:**
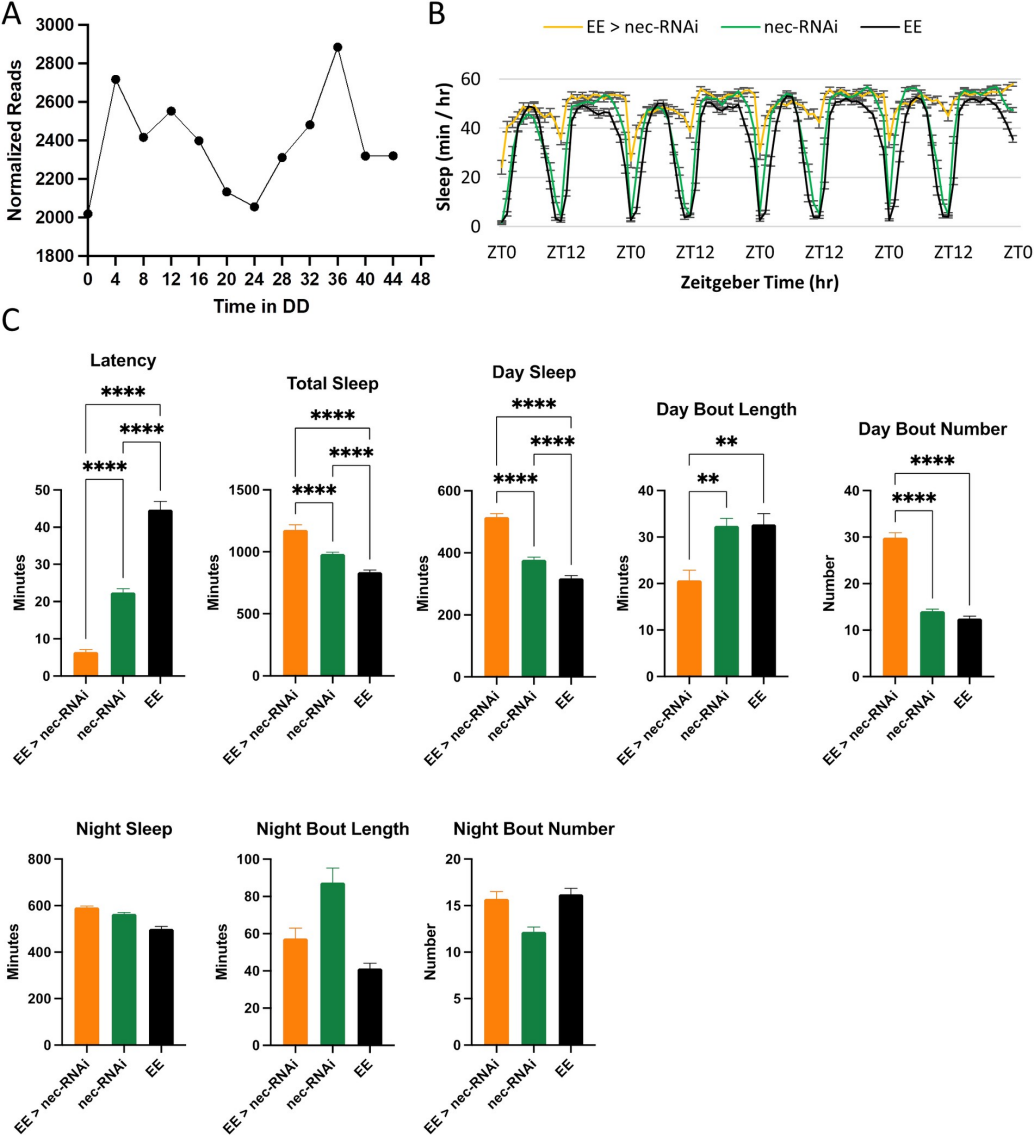
Astrocytic nec is required for normal day sleep. A) Normalized read count across two days in DD derived from the astrocyte TRAP. Results are averages of two data sets per time point. B) Average sleep behavior across four days for EE>nec-RNAi (orange, n = 56), nec-RNAi (green, n = 89), and EE (black, n = 87). C) Average latency, total sleep, day sleep, day bout length, day bout number, night sleep, night bout length and night bout number. Results are mean ± SEM, One-way ANOVA, *p < 0.05, ****p < 0.0001.

Both the Toll and Immune Deficiency (IMD) signaling pathways regulate the release of secreted factors, including BomBc2, as a consequence of an immune response. Our studies identified cycling genes encoding a number of Toll signaling components, including *spe*, and the *serpins Spn27A* and *nec*. Examination of all expressed astrocyte genes (S2 Table) revealed four additional Toll signaling components: *Toll-7*, s*pz*, *spz3*, *spz5*. Of the cycling genes, only astrocyte knockdown of *nec–*a negative regulator of Toll signaling–disrupted sleep patterns. Expression of either of two different RNAi transgenes targeting nec resulted in lethality or shortened life span, indicating a vital function. Despite the reduced lifespan, we obtained adult flies with normal morphology and seemingly normal locomotor behavior and were able to use them for sleep assays. Importantly, waking activity was normal for these flies suggesting that they were not simply debilitated (Day activity: knockdown, 2.71 ± 0.13 (counts/min); UAS, 2.73 ± 0.07 (counts/min); Gal4, 2.08 ± 0.06 (counts/min). Night activity: knockdown, 3.32 ± 0.16 (counts/min); UAS, 3.32 ± 0.10 (counts/min); Gal4, 2.58 ± 0.06 (counts/min)). Nonetheless, nec knockdown flies exhibited a robust increase in total sleep, due predominantly to increased day sleep (Fig 5B and 5C), and day sleep was also fragmented (Fig 5C). In contrast, night sleep was fairly normal. We note that latency to the first night bout was significantly shortened (Fig 5B and 5C), consistent with the sleep phenotype. We also tested a third RNAi strain and found decreased day bout length compared to the controls (S5 Table), indicative of fragmentation.

**Fig 6:**
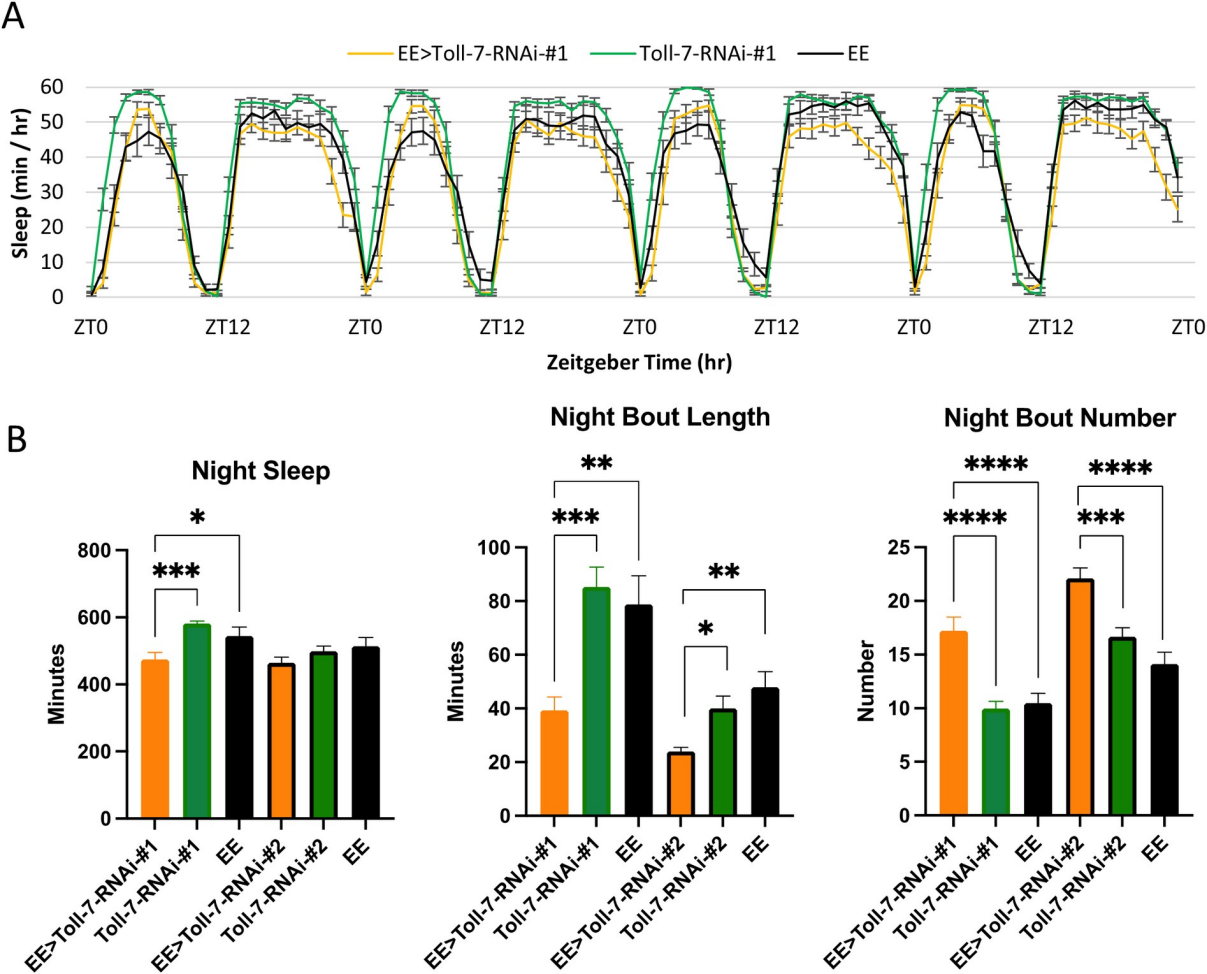
Knockdown of Toll-7 in astrocytes impairs night sleep. A) Average sleep behavior across four days for EE>Toll-7-RNAi-#1 (orange, n = 27), Toll-7-RNAi-#1 (green, n = 32), and EE (black, n = 24). B) Average night sleep, night bout length and night bout number for experiments with two different Toll-7-RNAi strains. For Toll-7-RNAi-#2, n = 22 (EE>Toll-7-RNAi-#2), n = 27 (Toll-7-RNAi-#2), n = 26 (EE). Results are mean ± SEM, One-way ANOVA, *p < 0.05, **p < 0.01, ***p < 0.001, ****p < 0.0001.

To more generally perturb Toll pathway signaling, we knocked down the *Toll-7* receptor, which our results demonstrate is expressed in astrocytes (S2 Table). Flies expressing Toll-7 RNAi#1 had significantly decreased night sleep (Fig 6A and 6B); expression of either RNAi#1 or RNAi#2 resulted in night sleep fragmentation (i.e., decreased night bout length and increased night bout number; Fig 6B) with no effect on day sleep (S5 Fig). Finally, we also perturbed IMD pathway signaling in astrocytes by knocking down PGRP-LC, a receptor that also mediates immune responses. In contrast to Toll-7, eaat1-Gal4>PGRP-LC knockdown flies had significantly decreased day sleep due to a decreased day bout duration (S6 Fig).

## Discussion

Although genome-wide expression profiles have been generated for astrocytes [21,41,42], this is the first study in either *Drosophila* or mammalian systems to describe the cycling transcriptome of astrocytes. Our studies identified 293 cycling genes; among these are core clock genes and those previously known to be expressed in astrocytes (Fig 2B and 2D and S3 Table). Surprisingly, comparison to a previous study of cycling genes in *Drosophila tim*-containing clock cells (both neurons and glia [17]), there was little overlap; only 34 genes, inclusive of the fly clock genes. This suggests that many cycling astrocyte RNAs are not rhythmically expressed in clock cells. Our experiments were conducted in constant environmental conditions (constant temperature and DD) and thus we think that many of the identified cycling genes are likely to be directly controlled by a PER-based clock. However, we cannot rule out the possibility that cycling may be due to indirect regulation such as changes in activity or metabolism.

Using Flybase, we identified mammalian orthologs of the 226 high confidence astrocyte-expressed cycling genes (those in the overlap of alrm and eaat1 expression patterns and which cycle in eaat1 expressing cells; S6 Table). We found mouse orthologs for 74% (167/226) and human orthologs for 73% (166/226) of the genes [43,44]. Of interest, mouse orthologs for 37 of the fly high confidence cycling genes are known to have enriched expression in mouse astrocytes [42] (S6 Table). These homologies underscore the value of the *Drosophila* model to study roles for astrocytes in nervous system function and behavior.

### Ribosome binding is restricted to particular times of day

The present study documented two major circadian peaks of ribosome-binding for our overall cohort of cycling RNAs (2E and 2F), suggesting temporally restricted translation for most transcripts. The previous TRAP analysis using tim-Gal4 also revealed two apparent phases of translation [17]. In both studies, peak ribosome binding occurred for many transcripts during mid-day or mid-night, periods of behavioral quiescence. In comparison, most genome-wide studies observed peaks of transcription/RNA abundance distributed throughout the entire cycle [45–49]. An exception to these studies was a recent study that performed expression profiling for different subgroups of clock neurons [50]; two overall phases of peak RNA abundance were observed in the PDF-positive ventral lateral neurons while a single peak was observed in the dorsal lateral neurons or dorsal neurons. Contrary to clock neurons, profiling of dopaminergic neurons in the same study did not show dominant phases of RNA abundance. Altogether, these studies suggest that a translational rhythm is layered upon the RNA abundance rhythm in many cell types, whereas certain clock neurons may have transcriptional/abundance rhythms with peaks restricted to particular times of the cycle. In our limited behavioral screen, we did not observe any correlation between peak times of ribosome-binding and effects on sleep. We do not find this surprising as the phases of ribosome-binding and the later action of an encoded protein would not necessarily be synchronous.

### Post transcriptional regulation

Post-transcriptional regulation of RNAs is a known method of control for circadian and sleep-related genes. Much has been written about the role of microRNAs (miRNAs) in the temporal control of cycling and we previously identified a number of miRNAs in astrocytes with essential functions in maintaining normal circadian behavior [51]. One less-discussed mechanism is nonsense-mediated decay (NMD), a highly conserved mechanism for removing transcripts with premature termination codons. While the primary function of this mechanism is to prevent aberrant proteins from being made, it is also known to participate in normal gene regulation [52]. We found that multiple genes encoding proteins of the NMD complex were expressed in astrocytes including Upf2, Upf3, Smg5, and Smg6 (S2 Table). Intriguingly, Smg6 was the only one found to cycle in our TRAP experiments (Fig 3A). SMG6 is a known endonuclease that cleaves NMD targets. A recent study showed that *Smg6* mutants have decreased behavioral rhythmicity, potentially through changing the ratio of cAMP response element-binding protein (CREB-B) isoforms [53]. In the current study, knockdown of Smg6 in glia, and specifically in astrocytes, significantly decreased and disrupted sleep at night (Fig 3, S3 Fig). Given the function of Smg6, it is possible that this sleep disruption is due to an increased amount of mistranslated protein. As the amount of Smg6 peaks in the early morning, it is possible that daytime accumulation of abnormal proteins is detrimental to night sleep (Fig 3A).

### Immune-related genes cycle in abundance and affect sleep

The features of sleep are similar in *Drosophila* and mammals [54], with sleep controlled by both circadian and homeostatic mechanisms. In flies and mammals, microbial infection affects sleep in the same way; infected flies have decreased sleep quality (fewer and shorter sleep bouts) before becoming arrhythmic [55]. Sleep deprivation in flies results in increased expression of many immune-related genes, including the NFκB homolog, *Relish* [56]. NFκB is an important regulator of immune response activated by Toll signaling and *Relish* was found to be essential for promoting sleep after injury or infection via the core circadian clock [57]. Innate immunity is under circadian regulation in both mammals and flies [58–62]. As a consequence, the *cyc*^*01*^ clock mutant has increased expression of immune-related genes [56], and fly *clock* mutants are immunocompromised, dying significantly faster after infection than wild-type flies [55].

Given the connection between immune/stress function and sleep, we focused on cycling genes relating to immunity.

We show that genes of the Turandot (*Tot*), Bomanin (*Bom*), and Growth Blocking Peptide-like (*Gbp*) immune/stress families are expressed in fly astrocytes, and in particular, *BomBc2*, *TotA*, *TotC*, *TotX*, *Gbp2 and Gbp3* exhibit rhythms in ribosome binding within this cell type (Figs 4A and S2B). Genes of these families mediate fly innate immunity and/or general responses to stress (oxidative stress, starvation, temperature changes or mechanical stress) [63–66]. The Turandots are a group of eight poorly characterized secreted peptides induced by sceptic injury. [63]. *TotA* activation occurs the JAK/STAT pathway but also requires the *Relish* pathway [38]. Knockdown of either *TotA* or *TotC* in astrocytes disrupted sleep (S4 Fig). In particular, astrocyte expression of TotA-RNAi decreased and fragmented day sleep (S4A and S4B Fig). The *Bomanin* family of genes consists of a dozen small secreted peptides that are activated in response to infection and required for Toll-mediated immune defense [67]. We identified BomBc2 (CG15067) as a novel cycling astrocyte gene (Fig 4A). Knockdown of BomBc2 in glia resulted in significantly decreased night sleep and increased day sleep, particularly in the first half of the day with a concomitant effect on sleep latency (Fig 4B and 4C). The effect on day sleep may represent a homeostatic response to decreased night sleep. Furthermore, night sleep was fragmented as there was a significant increase in night bout number and length (Fig 4C). We repeated these experiments using an astrocyte specific driver but did not see any effect on sleep, suggesting that this peptide may function primarily in another glial subtype.

Recent studies indicate that all components of Toll receptor signaling are expressed in the adult fly brain [68]. Thus, it is of interest that our results indicate expression of Toll signaling components in astrocytes, including *Toll-7*, s*pz*, *spz3*, *spz5*, *nec*, *Serpin 27A* and *spe*. Indeed, *Spe*, *nec*, *and Spn27A* mRNA exhibit rhythms in ribosome binding (S2B Fig and S2 Table) suggesting a circadian translational control of Toll signaling in astrocytes. *Spz*, an IL-1 analog, was recently described as a signaling mechanism for sleep pressure with sleep deprived flies exhibiting a 9-fold increases in astrocytic *spz* [40]. Knockdown of astrocyte *spz* reduced sleep recovery after sleep deprivation, whereas baseline sleep was not consistently affected.

In our studies, we asked whether additional astrocyte Toll signaling components are relevant for sleep. Of the cycling Toll components, only astrocyte knockdown of *nec* produced a sleep phenotype. *Nec* is a serine protease inhibitor that inhibits the cleavage of spz to a form that binds Toll. Expression of nec-RNAi in astrocytes resulted in increased day sleep, suggesting that increased mature spz may increase day sleep amount (Fig 5). In contrast, a recent study showed that day sleep was increased with astrocytic *spz* knockdown [40]. We also knocked down spz in astrocytes but did not find consistent effects between the two RNAi strains (S5 Table).

Finally, given our findings that immune factors regulate sleep, we perturbed two of the major immune signaling pathways of astrocytes: Toll and IMD. Our TRAP analysis showed that *Toll-7* is expressed in astrocytes, albeit not as a cycling gene (S2 Table), and knockdown of the gene decreased night sleep and caused sleep fragmentation (Fig 6). *PGRP-LC*, an IMD pathway receptor, is also expressed in astrocytes, and reduced PGRP-LC function resulted in decreased day sleep as a consequence of shorter day sleep bouts (S6 Fig), suggesting that the Toll and IMD pathways may differentially regulate night versus day sleep. Toll-7 and PGRP-LC genes do not cycle in abundance, but our studies, nonetheless, implicate them in sleep regulation, consistent with a role for these signaling pathways in this process. We recognize that either cycling or non-cycling genes may be critical for normal astrocyte physiology or morphology, with secondary effects on sleep, but it seems likely that certain cycling genes have important roles in the circadian regulation of sleep.

## Materials and methods

### Fly strains and maintenance

*Drosophila* cultures were reared on standard cornmeal/sugar/wheat germ medium in an environmentally controlled incubator set at 25°C on a 12 hr:12 hr light-dark (LD) schedule. For the isolation of astrocytes, virgin female flies carrying elav-Gal80, eaat1-Gal4 were crossed with male elav-Gal80, UAS-EGFP-L10a flies to generate F1 flies that express EGFP-L10a in astrocytes. For the RNAi screen, experimental flies were generated by crossing female repo-Gal4 flies with male flies containing the RNAi construct. For controls, both the driver and RNAi strains were crossed to w^1118^ flies.

### Purification and isolation of ribosome-bound RNAs

After an entrainment period of at least 4 days, flies were collected at 12 times points (every 4 hours) across a 48-hour dark period into 15 mL conical tubes before flash-freezing in liquid nitrogen. Flies were then stored at -80°C until further processing. Heads were separated by quickly vortexing the tubes and then using sieves to collect heads. There were ~200 heads per TRAP experiment sample. Frozen heads were quickly homogenized in buffer containing 20 mM HEPES-KOH (pH 7.4), 150 mM KCl, 5 mM MgCl_2_, 0.5 mM DTT, 100 μg/ml Cycloheximide and 2 U/mL SUPERase. Samples were then centrifuged at 20,000 x *g* for 20 minutes. The cleared lysate was separated from the debris into a new tube and DHPC and Igepal CA-630 was added to the solution at a concentration of 30 mM and 1%, respectively. Samples were incubated on ice for 5 minutes and were then centrifuged again at 20,000 x *g* for 20 minutes. After centrifugation, the supernatant was combined with magnetic beads coated with purified anti-EGFP antibody (HtzGFP-19C8, Sloan Kettering Antibody & Bioresource Core Facility; Dynabeads Antibody Coupling Kit, Invitrogen) and incubated at 4°C for 1 hour with end over end rotation. Samples were then washed 6 times with 20 mM HEPES-KOH (pH 7.4), 150 mM KCl, 5 mM MgCl_2_, 0.5 mM DTT, 100 μg/ml Cycloheximide. RNA was extracted from the magnetic beads using TRIzol. RNA quality and quantity were analyzed using a Bioanalyzer (Agilent).

### RNA-Seq library construction

Approximately 400 ng of RNA from each time point was used for the construction of cDNA libraries for RNA-Seq by following standard Illumina protocols from the TruSeq RNA sample preparation kit. mRNAs were isolated from ribosomal and other small RNAs using poly-dT coupled magnetic beads and fragmented by addition of divalent cations at 94°C. Cleaved mRNAs were then reverse transcribed into cDNA using random primers, and cDNA was subjected to second strand synthesis using DNA polymerase I and RNaseH. DNAs were end repaired, “A” tailed, and then ligated to Illumina sequencing adaptors prior to enrichment by PCR to create a library. The quality and quantity of each library was assessed using a Bioanalyzer or a Fragment Analyzer.

### RNA-seq analysis and identification of rhythmic mRNAs

100-bp single-end reads were obtained via an Illumina HiSeq2000 located in the Tufts Medical School Genomics core facility. On average approximately 27 million reads were obtained per biological replicate. FastQC (http://www.bioinformatics.babraham.ac.uk/projects/fastqc/) was used to assess read quality. The mean quality score obtained for all sequencer runs was 36.2. Before alignment, adapter sequences were removed using FastX 0.0.13 (http://hannonlab.cshl.edu/fastx_toolkit/index.html). Reads were aligned to the BDGP Release 6 genome assembly [69] using Tophat 2.0.9 [70] and Bowtie 2.1.0 [71]. On average, 73% of reads for replicate 1 were mapped and 86% of reads were mapped for replicate 2. For the reads that were successfully mapped, on average 90.6% mapped uniquely for replicate 1 and 91.7% of reads were uniquely mapped for replicate 2. Numbers of reads aligning to annotated genes were tallied using HTSeq-count [72]. Counts produced by HTSeq were updated to gene IDs downloaded from FlyBase version FB2017_1 via a Python-based sqlite script. Genes with average read counts of less than 20 across all timepoints were removed and the count numbers were quantile normalized to account for variation in raw read counts across timepoints. Scatter plots of read numbers between replicates and regression analysis was carried out in R (3.6.2). Genes were annotated using the Database for Annotation, Visualization and Integrated Discover (DAVID, v. 6.8) and FlyBase (FB2019_6) [37,44]. Circadian cycling was assessed using JTK_CYCLE [34]. Genes were considered significant if the cycling amplitude was greater than 0.5 and the adjusted p value was greater than 0.05.

### Behavior assays

Male flies (< 1 week old) were loaded into Drosophila activity monitors (DAMs, Trikinetics) placed in environmentally controlled incubators kept at 25°C, 12:12 LD. Locomotor activity for sleep was collected in 1-minute bins with 5 ≥ minutes of immobility considered the cutoff for sleep. Analyses of sleep behavior were performed using an Excel-based package [73]. References in the text to day and night sleep, respectively, refer to ZT 1–11 and ZT 13–23. Latency refers to the time until the first night sleep bout after lights-off. Circadian locomotor activity was collected in 30-minute bins and analyzed using a MATLAB-based signal processing flu toolbox [74]. To assess the statistical significance of locomotor data, normality was determined using the D’Anostino and Pearson omnibus normality test. If the data did not pass a normality test, we used the Kruskal-Wallis test (nonparametric ANOVA) with Dunn’s multiple comparison test. If normality was observed, we used a one-way ANOVA with the Tukey-Kramer multiple comparisons test.

### Immunohistochemistry

At CT0, flies were fixed in 4% paraformaldehyde (PFA) in PBST (1 x PBS, 0.5% Triton-X-100) on ice for 30 minutes. Brains were dissected in PBST and then fixed in 4% PFA for 20 minutes. Fixed brains were washed with PBST, followed by blocking in 5% normal goat serum for 3 hours. Brains were incubated with primary antibody for 48 hours at 4°C, washed with PBST 6 times for 30 minutes each, and then incubated with secondary antibody overnight at 4°C. Brains were then washed with PBST 6 times before mounting with VectaShield (Vector Laboratories, Burlingame, CA). We used the following primary antibodies: mouse anti-REPO [1:500; Developmental Studies Hybridoma Bank (DSHB)] and rabbit anti-PER (1:10,000, a gift from R. Stanewsky). Relevant secondary antibodies were used at 1:500, including Alexa Fluor 488 goat anti-rabbit and Alex Fluor 647 goat anti-mouse (Invitrogen, Carlsbad, CA). Brain images were acquired using a Nikon A1R confocal microscope.

For the cell counting experiments shown in Figs 1 and 2, a series of 1 μm steps images were acquired using a 40x oil objective. Each stack of images was then divided into 6 μm thick substacks before consolidation into separate max-intensity images at which time one image per brain was chosen to use for counting. All stacks chosen were of similar anatomical location. ImageJ software was used to identify astrocytes (in green, Fig 1A or red and Fig 2D in the chosen stack. The identified cells were then assessed for REPO (Fig 1A) or PER (Fig 2D) positivity.

## Supporting information

S1 Fig Scatter plots comparing each sample across all 12 time points. Y and X–axes represent different samples. t = time in DD and r = correlation coefficient.
(TIF)

S2 Fig Normalized read counts for stress and immunity related genes. A) Heatmap showing normalized read counts for 13 cytochrome P450 family members. Yellow is high abundance and blue is low abundance. Results are averages of two data sets per time point. B) Normalized read count across two days in DD for Turandots and Growth-Block Peptides C) Normalized read count across two days in DD for Toll pathway components SPE and Spn27A.
(TIF)

S3 Fig Astrocyte knockdown of SMG6 does not affect day sleep. Average latency, day sleep, day bout length and day bout number for REPO (A) and EE (B) experiments depicted in Fig 3.
(TIF)

S4 Fig Astrocyte knockdown of TotA and TotC disrupts sleep. A) Average sleep behavior across four days for EE>TotA-RNAi (orange, n = 30), TotA-RNAi (green, n = 32), and EE (black, n = 24). B) Average total sleep, day sleep, day bout length, day bout number, night bout length and night bout number. Results are mean ± SEM, One-way ANOVA, *p < 0.05, **p < 0.01, ***p < 0.001, ****p < 0.0001. C) Average sleep behavior across four days for EE>TotC-RNAi (orange, n = 28), TotC-RNAi (green, n = 26), and EE (black, n = 24). D) Average total sleep, day sleep, day bout length, day bout number, night bout length and night bout number. Results are mean ± SEM, One-way ANOVA, *p < 0.05, **p < 0.01, ***p < 0.001, ****p < 0.0001.
(TIF)

S5 Fig Astrocyte knockdown of Toll-7 does not affect day sleep. Average latency, total sleep, day sleep, day bout length and day bout number for Toll-7 RNAi-#1(A) and -#2 (B).
(TIF)

S6 Fig Astrocyte knockdown of PGRP-LC disrupts day sleep. A) Average sleep behavior across four days for EE>PGRP-LC-RNAi (orange, n = 32), PGRP-LC-RNAi (green, n = 32), and EE (black, n = 30).
(TIF)

S1 Table RNA-Seq Stats Summary. (XLSX)

S2 Table All Detected Genes. (XLSX)

S3 Table Cycling Astrocyte Genes. (XLSX)

S4 Table Previously Identified Cycling Genes. (XLSX)

S5 Table Astrocyte RNAi Sleep Screen. (XLSX)

S6 Table Mammalian Homologs for Cycling Genes. (XLSX)
