# Supplementary figures and images for: Circadian regulation of the Drosophila astrocyte transcriptome
Source: PLoS Genet. 2021 Sep 20;17(9):e1009790. doi: 10.1371/journal.pgen.1009790 (PMC8483315; doi:10.1371/journal.pgen.1009790)

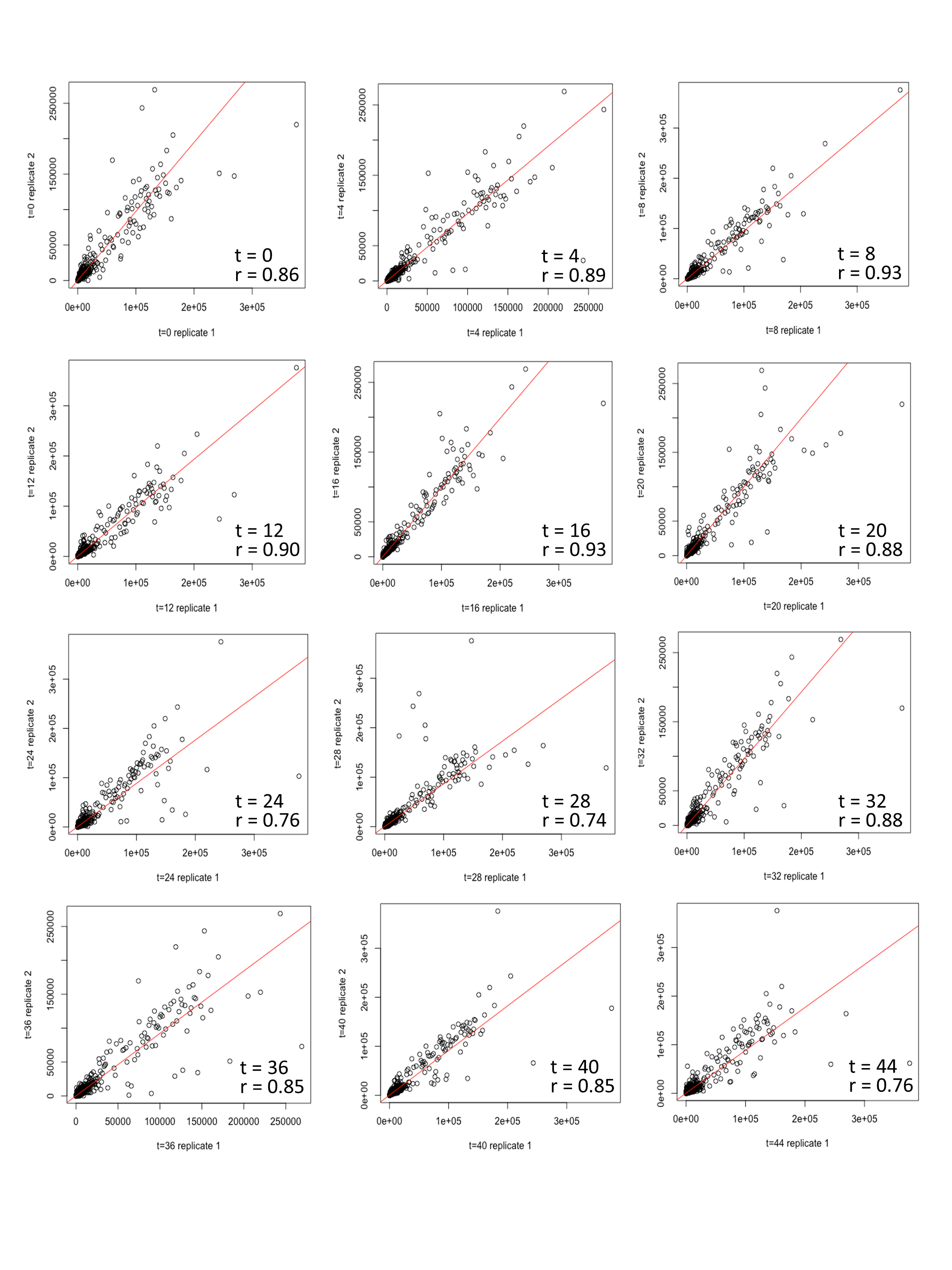

Supplement: S1 Fig — Y and X–axes represent different samples. t = time in DD and r = correlation coefficient. (TIF) [file pgen.1009790.s001.tif]

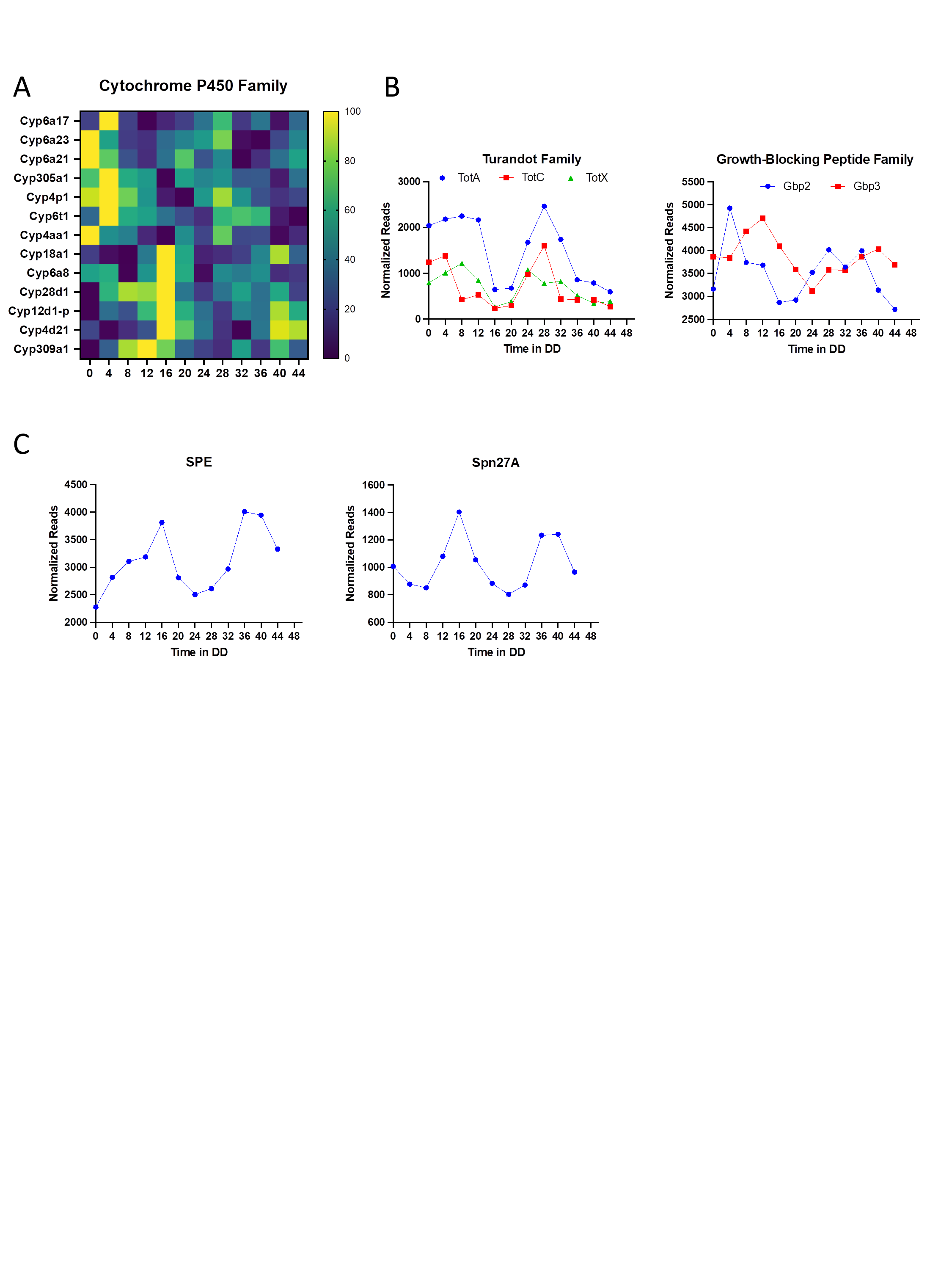

Supplement: S2 Fig — A) Heatmap showing normalized read counts for 13 cytochrome P450 family members. Yellow is high abundance and blue is low abundance. Results are averages of two data sets per time point. B) Normalized read count across two days in DD for Turandots and Growth-Block Peptides C) Normalized read count across two days in DD for Toll pathway components SPE and Spn27A. (TIF) [file pgen.1009790.s002.tif]

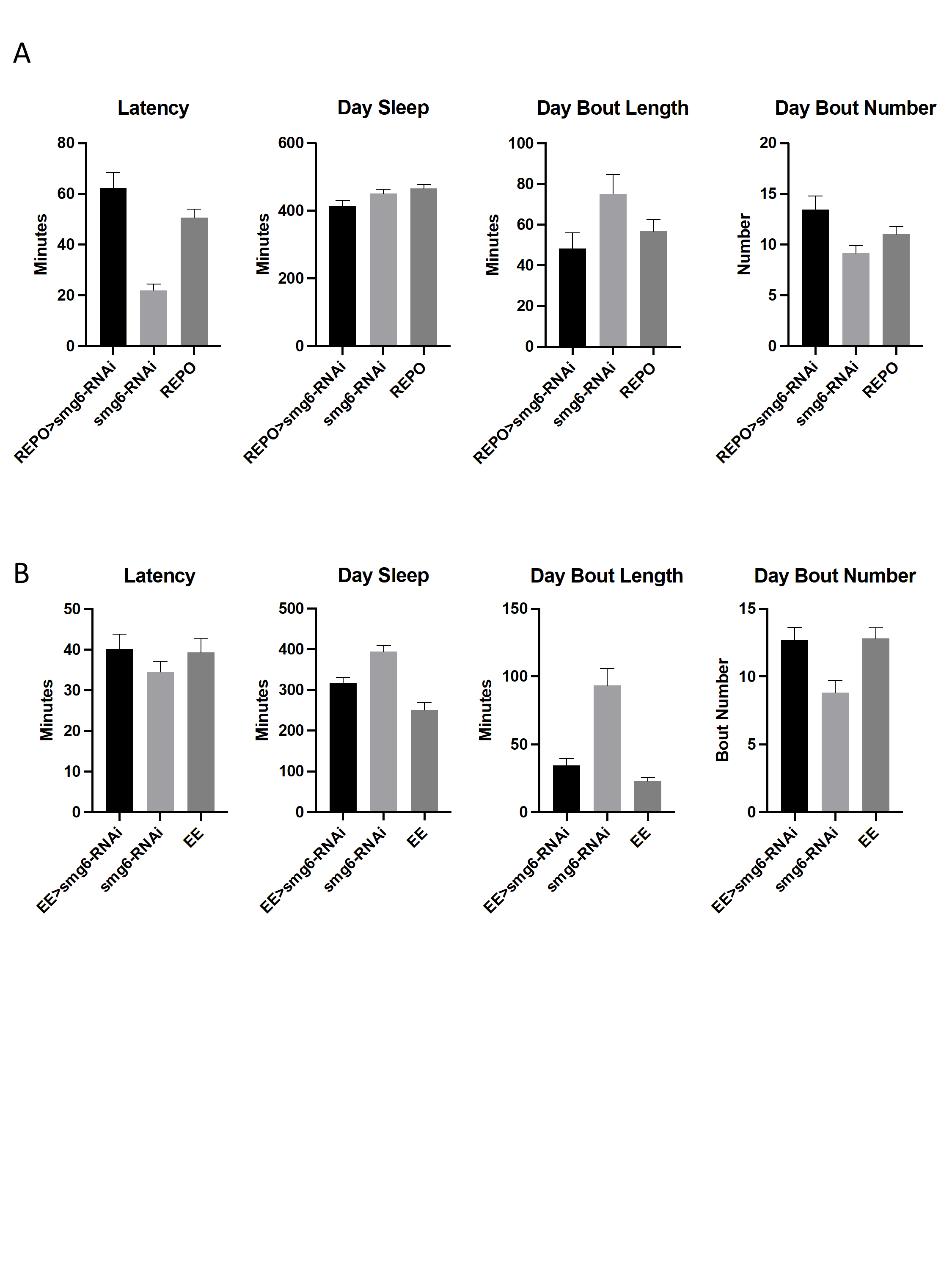

Supplement: S3 Fig — Average latency, day sleep, day bout length and day bout number for REPO (A) and EE (B) experiments depicted in Fig 3. (TIF) [file pgen.1009790.s003.tif]

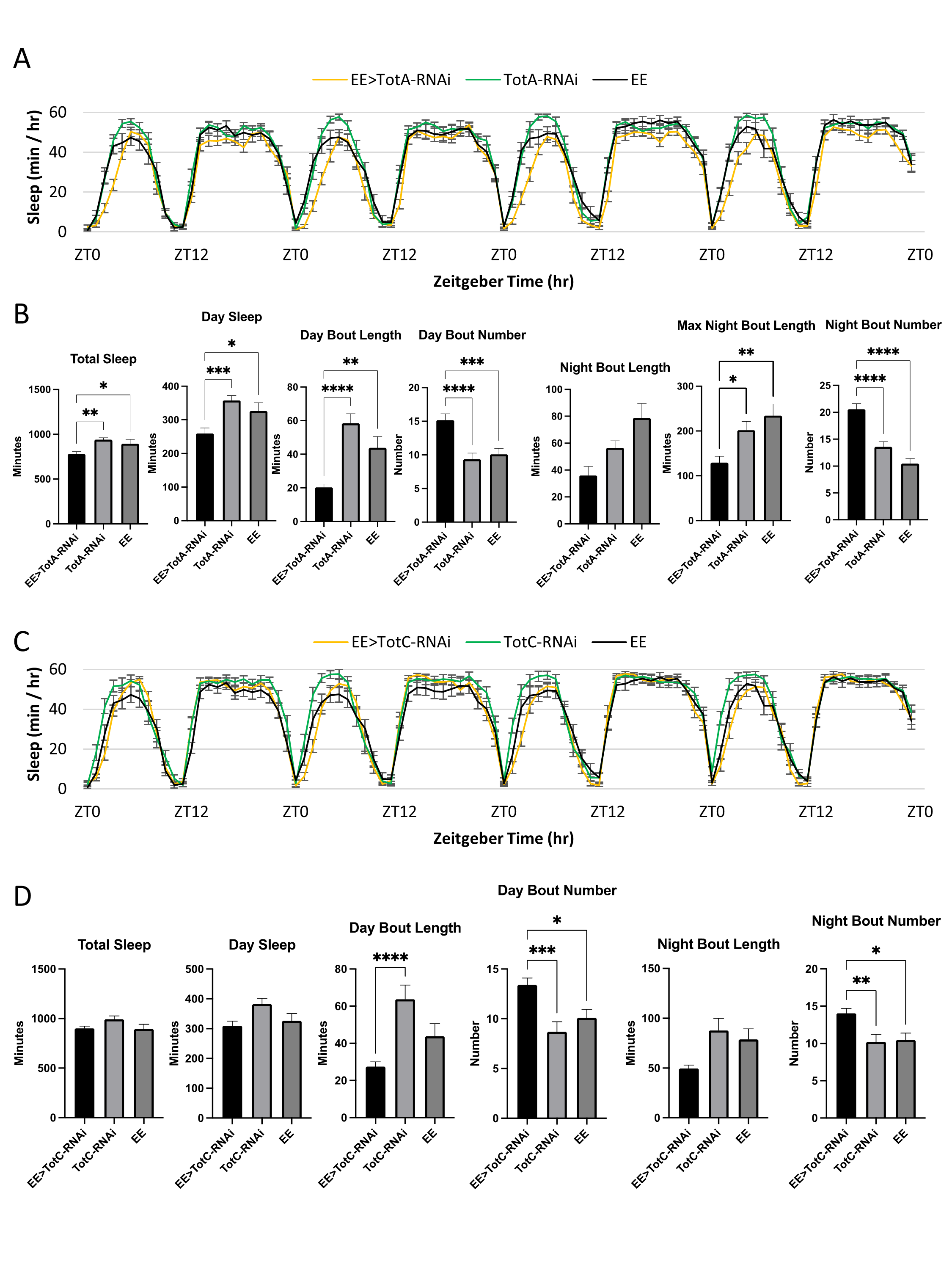

Supplement: S4 Fig — A) Average sleep behavior across four days for EE>TotA-RNAi (orange, n = 30), TotA-RNAi (green, n = 32), and EE (black, n = 24). B) Average total sleep, day sleep, day bout length, day bout number, night bout length and night bout number. Results are mean ± SEM, One-way ANOVA, *p < 0.05, **p < 0.01, ***p < 0.001, ****p < 0.0001. C) Average sleep behavior across four days for EE>TotC-RNAi (orange, n = 28), TotC-RNAi (green, n = 26), and EE (black, n = 24). D) Average total sleep, day sleep, day bout length, day bout number, night bout length and night bout number. Results are mean ± SEM, One-way ANOVA, *p < 0.05, **p < 0.01, ***p < 0.001, ****p < 0.0001. (TIF) [file pgen.1009790.s004.tif]

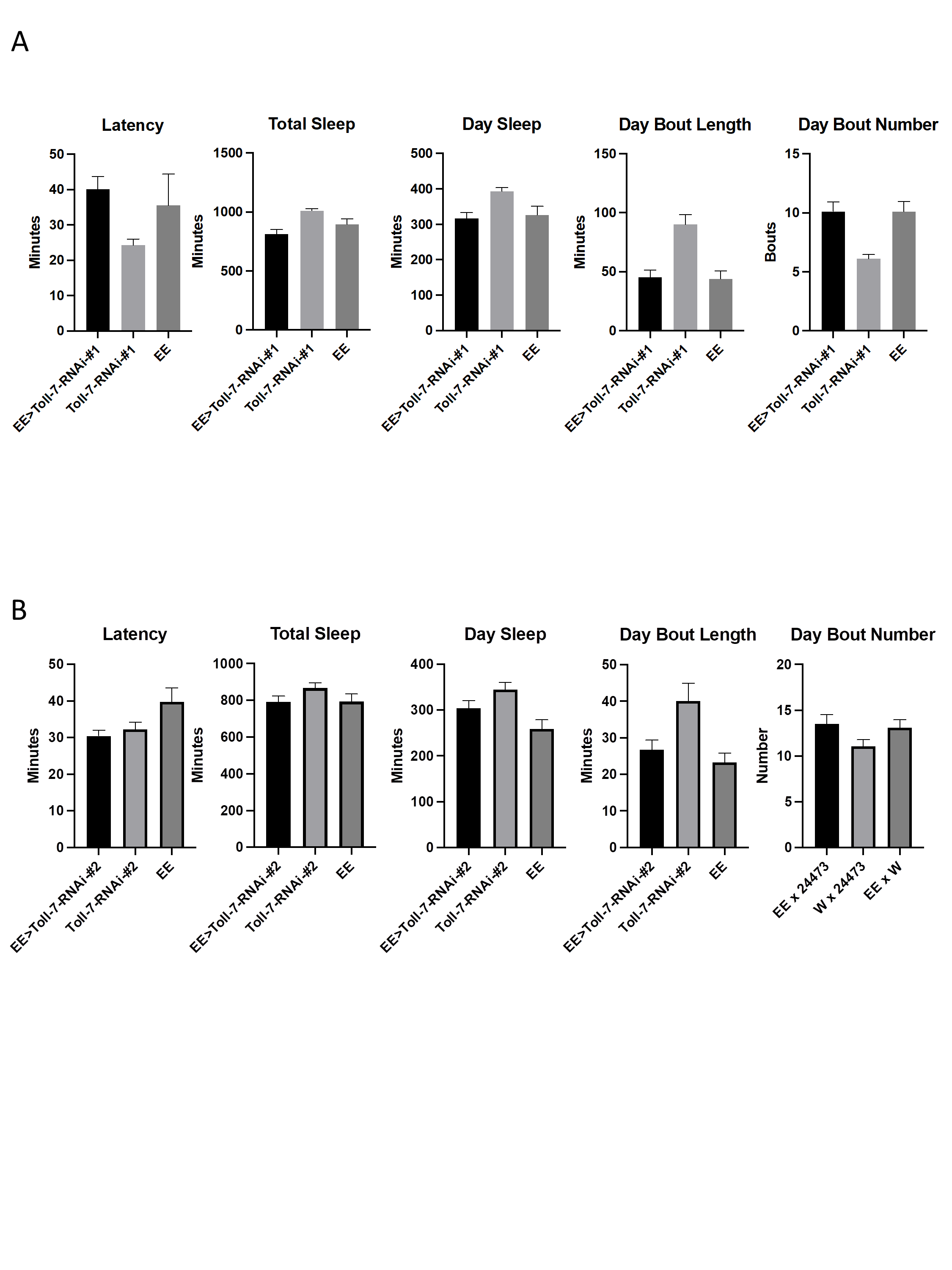

Supplement: S5 Fig — Average latency, total sleep, day sleep, day bout length and day bout number for Toll-7 RNAi-#1(A) and -#2 (B). (TIF) [file pgen.1009790.s005.tif]

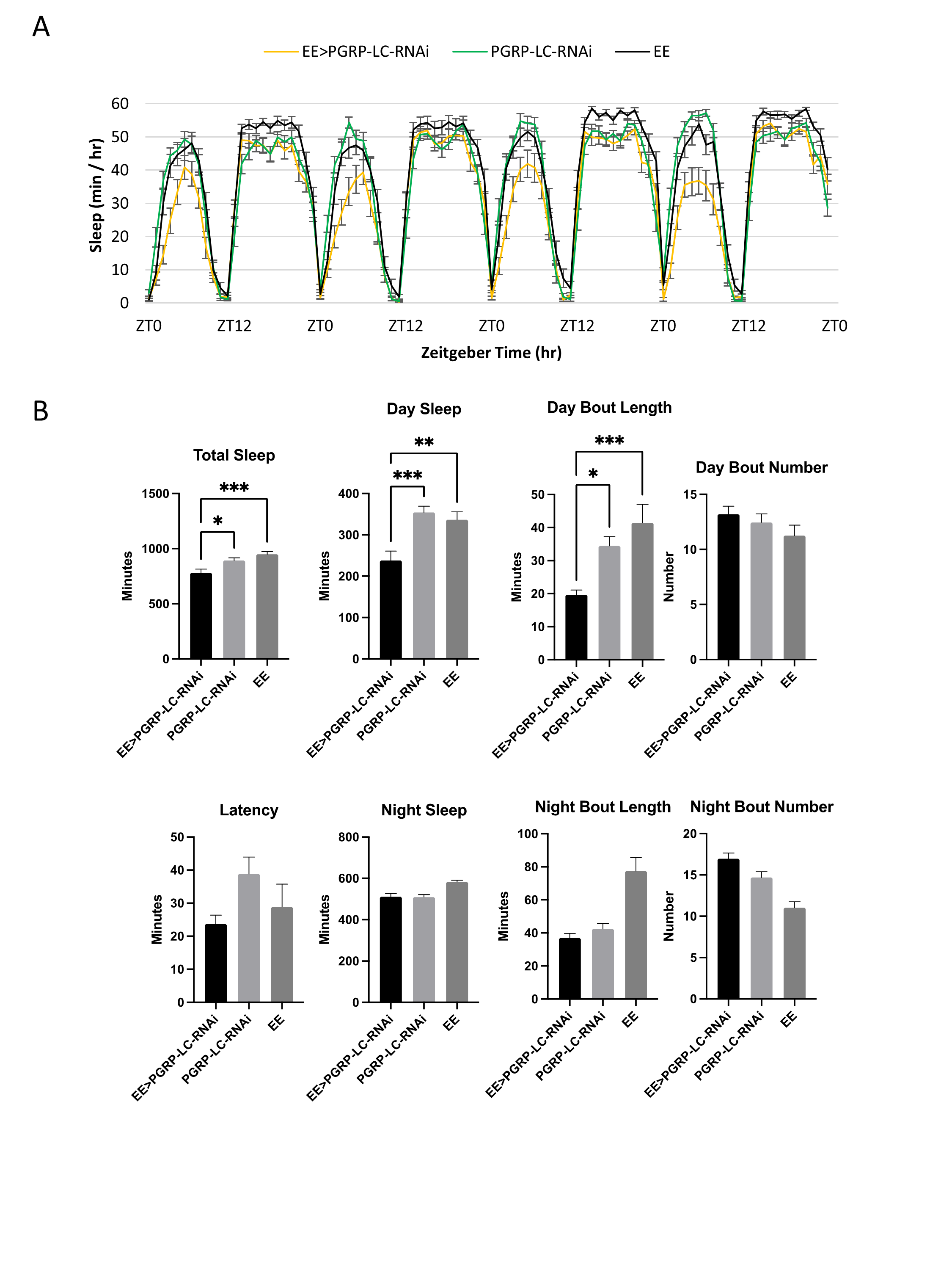

Supplement: S6 Fig — A) Average sleep behavior across four days for EE>PGRP-LC-RNAi (orange, n = 32), PGRP-LC-RNAi (green, n = 32), and EE (black, n = 30). (TIF) [file pgen.1009790.s006.tif]
